# The Role of the Canadian Media During the Initial Response to the COVID-19 Pandemic: A Topic Modelling Approach Using Canadian Broadcasting Corporation News Articles

**DOI:** 10.2196/25242

**Published:** 2021-07-18

**Authors:** Janhavi Patel, Harsheev Desai, Ali Okhowat

**Affiliations:** 1 Michael G DeGroote School of Medicine McMaster University Hamilton, ON Canada; 2 Faculty of Engineering and Architectural Science Ryerson University Toronto, ON Canada; 3 UBC Global Health Faculty of Medicine University of British Columbia Vancouver, BC Canada

**Keywords:** COVID-19, topic modelling, LDA, health communication, mass media, coronavirus, media, dissemination, online health information, public health

## Abstract

**Background:**

Beginning as a local epidemic, COVID-19 has since rapidly evolved into a pandemic. As countries around the world battle this outbreak, mass media has played an active role in disseminating public health information.

**Objective:**

The aim of this study was to get a better understanding of the role that the Canadian media played during the pandemic and to investigate the patterns of topics covered by media news reporting.

**Methods:**

We used a data set consisting of news articles published on the Canadian Broadcasting Corporation (CBC) website between December 2019 and May 2020. We then used Python software to analyze the data using Latent Dirichlet Allocation topic modelling. Subsequently, we used the pyLDAvis tool to plot these topics on a 2D plane through multidimensional scaling and divided these topics into different themes.

**Results:**

After removing articles that were published before the year 2019, we identified 6771 relevant news articles. According to the CV coherence value, we divided these articles into 15 topics, which were categorized into 6 themes. The three most popular themes were case reporting and testing (n=1738), Canadian response to the pandemic (n=1259), and changes to social life (n=1171), which accounted for 25.67%, 18.59%, and 17.29% of the total articles, respectively.

**Conclusions:**

Understanding the Canadian media’s reporting on the COVID-19 pandemic shows that the Canadian pandemic response is a product of consistent government communication, as well as the public’s understanding of and adherence to protocols.

## Introduction

COVID-19, which started as a local epidemic, evolved into a pandemic in a matter of months [1]. Countries around the world are battling the spread of this disease and the unfortunate consequences of COVID-19–related mortality and morbidity, resource limitations, and severe economic burden [1,2]. Canada is no different and continues to observe a rising number of COVID-19 cases [3].

Due to the initial lack of vaccines and knowledge about the disease and its treatment, countries were forced to take unique approaches to combat the spread of the virus. Canada's response has been widely reported as being adequate, though much more could have been and remains to be done in tackling the spread of COVID-19 [4]. The Canadian government website for its COVID-19 response highlights the measures Canada has taken, including the creation of the COVID Alert app, an ethics framework for policy makers, and economic support for Canadians. Support involves both financial measures and safety, including for Canadians abroad and vulnerable populations in Canada [3]. Additionally, there has been an emphasis on public education, collaboration, and guidance for researchers and frontline health workers [3].

With the uncertainty surrounding this novel coronavirus, the media—especially online news sources—have played a key role in informing the public about events related to the pandemic. Mass media has been successfully used for decades to increase public health awareness. News media outlets have been used across the globe for addressing public health issues like reducing tobacco use, participating in screening for cancer, and cardiovascular disease prevention [5]. The Eat Well campaign, which was advertised through a combination of news and commercial media outlets, increased awareness about meal prepping and healthy food choices in the Canadian population [6]. A postcampaign evaluation showed that low-resource communities had a greater uptake of information, thus highlighting the need to better understand the impact of different information dissemination campaigns to better cater to the target population [6].

The Canadian Broadcasting Corporation (CBC) is a daily source of local and national information for many Canadians [7]. The CBC’s digital offering sees an average of 16.1 million new monthly visits [7] and continues to grow every month. Assessing the content of CBC articles can therefore provide insights into the information delivered to Canadians about the pandemic. Given that success in the fight against the pandemic greatly depends on the support of the public (eg, maintaining appropriate social distance and taking proper precautions), the information that media outlets report is important as it provides the public with up-to-date guidance.

The aim of this study was to better understand the role that news articles played in disseminating public health information, by specifically focusing on the topics reported and frequency of each topic reported regarding the COVID-19 pandemic. The methods used and results from this study could be relevant when reporting future events related to health care and national safety, which rely heavily on public support and awareness.

## Methods

### Data Collection

The data set was collected from the CBC website using a Python programming language script [8]. The script was used to extract information from over 6700 news articles, including the title, article summary, and main text for each article, using the term “coronavirus” as the search word. The extracted news articles were published between December 2012 and May 2020; however, only the articles published in 2019 and 2020 were included in this study.

We used Latent Dirichlet Allocation (LDA) to analyze these news articles. LDA is a three-level hierarchical Bayesian model, in which each item of a collection is modelled as a finite mixture over an underlying set of topics. The basic idea behind LDA is that documents can be represented as arbitrary mixtures over latent topics, which in turn are characterized by a distribution over words [9]. LDA has been extensively used and evaluated for its applicability in topic modelling research [10,11]. Moreover, Lancichinetti [12] showed that LDA has high reproducibility and accuracy for topic classification.

According to LDA, there are diverse topics in each news article, and the words in these articles can be allotted to one of these topics. However, LDA only groups inputs (ie, news articles in this case) based on the abovementioned distribution over words and it is subjective how these groups are interpreted as topics. To facilitate accurate representation, randomly selected articles from each topic were manually checked to make sure they were consistent with the interpreted topic.

### Data Processing

There were a total of 6771 news articles remaining after removing the articles published before 2019. These remaining articles were dated between December 22, 2019, and May 3, 2020.

**Figure 1:**
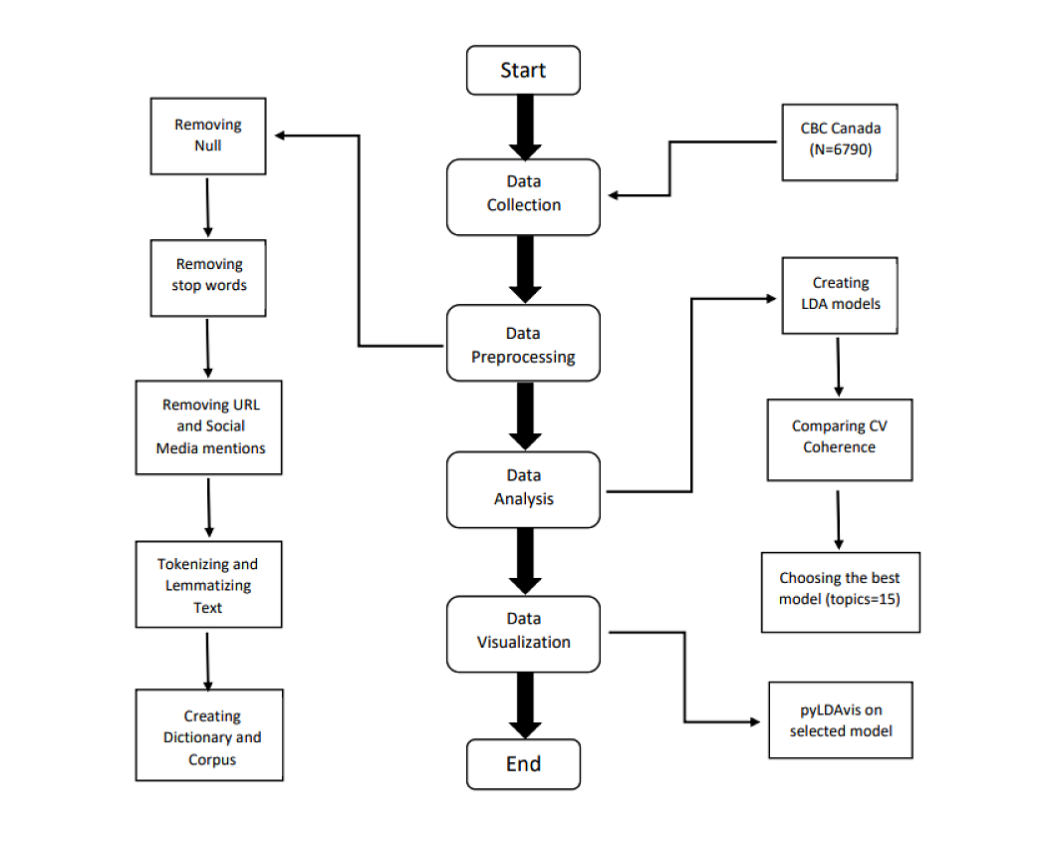
Workflow chart. CBC: Canadian Broadcasting Corporation; LDA: Latent Dirichlet Allocation.

Before moving forward with topic modelling, we used Python along with libraries, including the Pandas and Natural Language Toolkit (NLTK) libraries [13], to clean the data. The detailed process for this is displayed in Figure 1. We used the English language stop words provided by NLTK to remove common words such as “an,” “all,” “and,” “for,” and “from” as they hold no semantic value for our analysis. URLs and social media mentions consisting of “@” were also removed. The two primary inputs to the LDA model are the dictionary and the corpus, which were created using the Gensim library [14].

We used the CV coherence score to evaluate models with different numbers of topics and selected the one with the highest CV score. This approach mitigates one of LDA’s limitations—the need to know the number of topics ahead of time. According to Röder et al [15], the CV coherence score is one of the fastest measures of coherence, and the most accurate. Henri Trenquier defines coherence as the human's semantic appreciation of a topic represented by its N top words [16]. We chose the top 15 (N) words in each topic to calculate the coherence.

**Figure 2:**
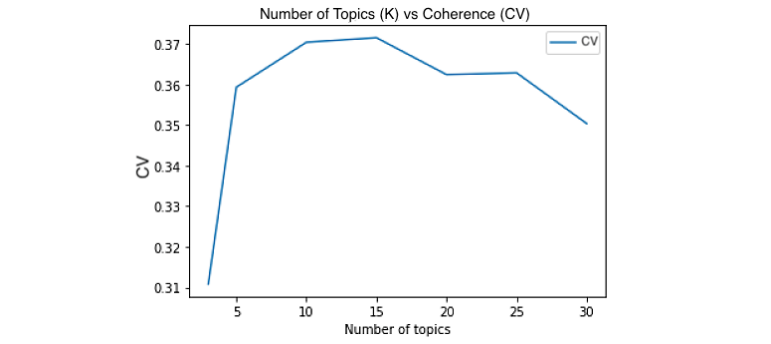
Coherence score for number of topics.

As evident from Figure 2, the highest CV coherence score was achieved at 15 topics. This means that the top words in each topic were most closely related semantically when the news article data set was divided into 15 different topics using LDA.

**Figure 3:**
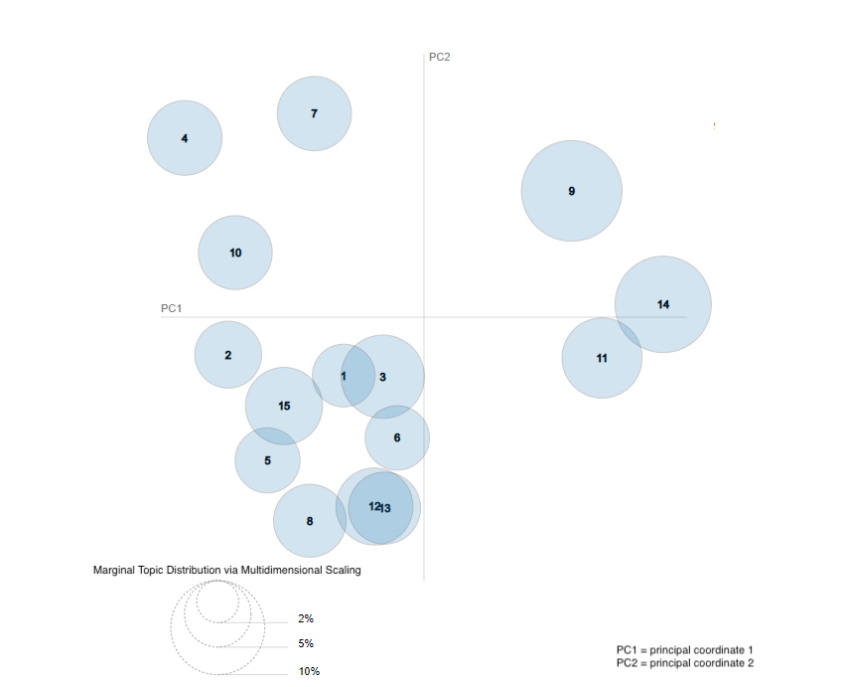
Intertopic distance map.

We then used the pyLDAvis tool [17] and Python to further analyze the 15 topics to extract valuable insights from the articles. The 15 topics were represented on an intertopic distance map, which is an interactive representation offered by the pyLDAvis tool (Figure 3). The topics are plotted as circles in a 2D plane whose centers are determined by computing the distance between topics [16].

**Figure 4:**
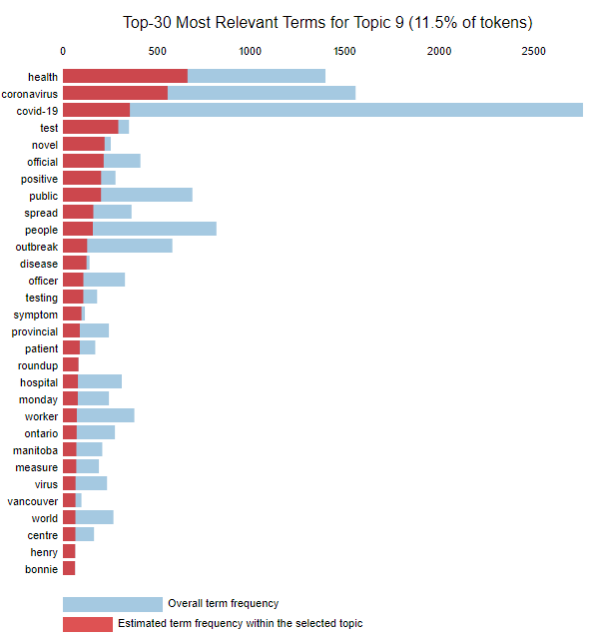
Top 30 most relevant terms (λ=1.0).

**Figure 5:**
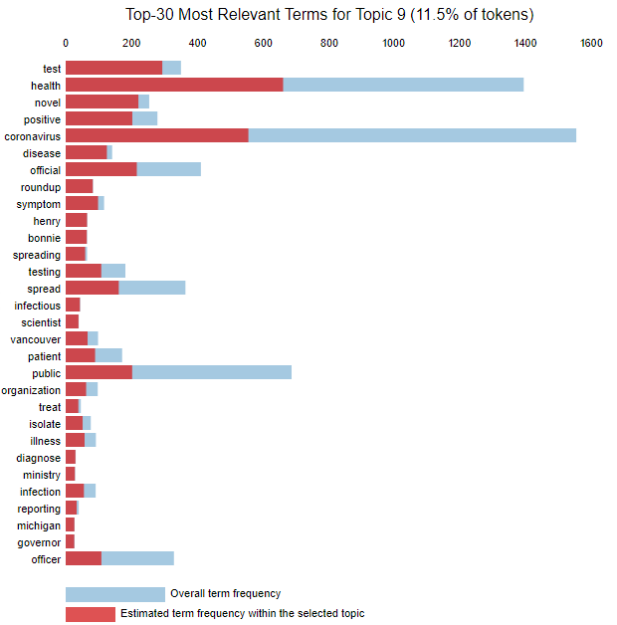
Top 30 most relevant terms (λ=0.4).

The weight parameter λ was adjusted to find the theme for each topic based on the top words in the topic. Setting λ=1 ranks the words in a topic by frequency, while setting λ=0 ranks the words based on uniqueness to that topic [17]. We used the interactive bar provided by the pyLDAvis tool to adjust λ and understand the theme for each of the 15 topics. To use topic 9 as an example, Figure 4 shows the top 30 most frequently occurring words in topic 9. As multiple topics might have similar words that occur frequently, we need to adjust the λ value to better gauge what topic an article might be about. For instance, when we set λ to 0.04, the terms most unique to topic 9 are captured, and presented in descending order in Figure 5. This analysis identifies words like “test,” “positive,” and “spread” as being unique to topic 9. Using the keywords, we identified that the general theme of articles in topic 9 is “testing.” This process was repeated for each topic (Table 1).

**Table 1:** Themes and topics (N=6771).

Themes and topics		Number of news articles, n (%)^a^	Keywords
**Theme 1: Case reporting and testing (n=1738)**			
	Topic 9: Testing	876 (12.97)	Coronavirus, spread, public, positive, novel, official, people, health, test, covid
	Topic 14: Case reporting	862 (12.73)	Province, number, confirm, total, people, report, health, death, case, covid
**Theme 2: Canadian response to pandemic (n=1259)**			
	Topic 5: General response	295 (4.37)	Pandemic, nation, member, Windsor, covid, community, family, first, local, want
	Topic 6: Health care/hospital response	267 (3.93)	Emergency, temporary, staff, state, hospital, Sudbury, worker, declare, general, covid
	Topic 10: Vaccine research	399 (5.88)	Ottawa, world, around, Canada, global, point, latest, covid, coronavirus, point
	Topic 1: Medical supplies and resources	298 (4.40)	Canadian, ventilator, doctor, could, Canada, happening, mask, province, available, covid
**Theme 3: Changes to everyday life (n=1171)**			
	Topic 2: Social gathering cancellations	322 (4.76)	Summer, pandemic, coronavirus, cancel, festival, university, event, season, covid, plant
	Topic 8: School closure/virtual learning	397 (5.88)	Parent, school, family, child, learning, student, covid, equipment, worker, pandemic
	Topic 12: General lifestyle changes	452 (6.68)	People, avoid, coming, together, change, normal, covid, province, pandemic, government
**Theme 4: Communication from the government (n=1002)**			
	Topic 11: Public health announcements	481 (7.04)	Medical, chief, public, officer, health, people, province, covid, Friday
	Topic 3: Prime Minister’s addresses	521 (7.68)	Minister, prime, Justin, Trudeau, worker, pandemic, essential, coronavirus, health, covid
**Theme 5: International news (n=826)**			
	Topic 4: Articles related to news in the United States	388 (5.73)	Trump, unite, outbreak, state, president, country, cruise, coronavirus, Canada, Canadian
	Topic 7: Articles related to news in China	438 (6.48)	Chinese, china, outbreak, answer, Canadian, flight, morning, expert, question, expert
**Theme 6: Government initiatives (n=775)**			
	Topic 13: Initiatives for vulnerable populations	343 (5.08)	Shelter, homeless, social, distance, encourage, people, pandemic, covid, measure, physical
	Topic 15: Economy and business	432 (6.39)	Business, economy, government, federal, pandemic, support, covid, million, premier Canada

^a^The percentages have been calculated using the N value (ie, 6771).

## Results

**Figure 6:**
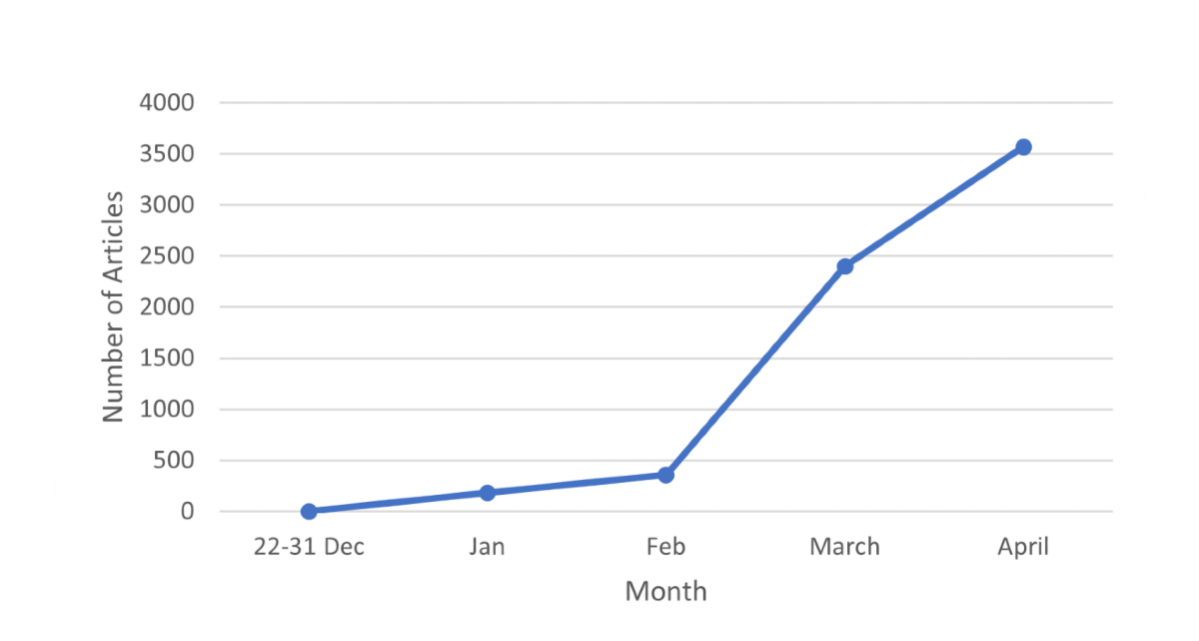
Time trend for number of articles.

Using the pyLDAvis tool, we grouped the 15 topics into 6 themes as shown in Table 1. Theme 1 (case reporting and testing) had the greatest number of articles (n=1738), while theme 6 (government initiatives) represented the lowest number of news reports (n=775). The trend in the total frequency of articles related to COVID-19 over our study time period is shown in Figure 6.

**Figure 7:**
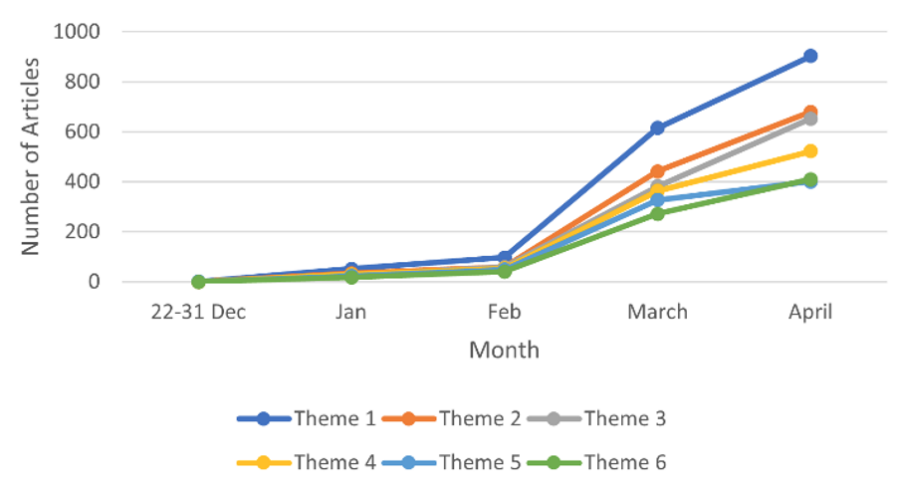
Time trends for each theme.

The most frequent theme, theme 1 (case reporting and testing), consisted primarily of topics that covered articles related to information about testing (12.97%) and case reporting (12.73%). The information in the articles related to testing focused on information regarding the tests being conducted to assess the spread of the virus, whereas the articles related to case reporting primarily focused on reporting the number of confirmed cases and deaths around the country, with words like “number,” “report,” “confirm,” and “death” being frequently used. Similar to the trend in frequency of total articles about COVID-19, Theme 1 saw a sudden increase in the number of articles published, starting from the month of February and increasing throughout March and April (Figure 7).

The Canadian media's focus in relation to the outbreak and Canada’s response is highlighted in themes 2 and 3. Theme 2 (Canadian response to pandemic) includes topics like general response (topic 5, n=295, 4.37%), health care/hospital response (topic 6, n=267, 3.93%), vaccine research (topic 10, n=399, 5.88%), and medical supplies and resources (topic 1, n=298, 4.40%). Theme 3 (changes to everyday life) discusses the changes resulting from the pandemic and includes topics like social gathering cancellations (topic 2, n=322, 4.76%), school closure/virtual learning (topic 8, n=397, 5.88%), and general lifestyle changes (topic 12, n=452, 6.68%). Both these themes saw a steep increase in the number of reported articles after the month of February.

Theme 5 (international news) consisted primarily of topics related to the United States (n=388, 5.73%) and China (n=438, 6.48%). The information in articles related to the United States, Canada's geographic neighbor and largest trading partner, focused more on political relations, with frequently used words like “president,” “Trump,” and “state.” On the contrary, the articles about China, the place of origin of the coronavirus, primarily focused on information that would enable a better understanding of the outbreak, with words like “outbreak,” “question,” and “answer” used more frequently.

Themes 4 and 6, although not high in frequency, focused on important themes like communication from the government and government initiatives. Communication from the government included both public health announcements as well as the Prime Minister's addresses to the public. The government initiatives theme included topics that discussed initiatives for vulnerable populations, specifically people experiencing homelessness (topic 13, n=343, 5.008%), as well as the economy and business (topic 15, n=432, 6.39%). The articles about government announcements had a steep increase leading to the declaration of pandemic in March 2020 but the slope reduced from March to April. Contrastingly, theme 6 saw a steep, consistent increase from February to April 2020.

## Discussion

### Principal Results

The COVID-19 pandemic has been a steep learning curve for all countries worldwide. Dissemination of information in a timely manner across communities and countries was crucial to limit the spread of COVID-19 and determine the efficacy of different treatment and management interventions. With the ensuing social isolation, online media took over as an important source of information available to the public; thus, understanding the role that the media played highlights key aspects of the challenges faced by Canada and its response to the pandemic. We used topic modelling using articles collected from the CBC's online platform and identified different themes reported through the articles.

Even though some reports about a fatal pneumonia of unknown cause had started coming out from China in early January, it was not until after the World Health Organization declared COVID-19 as a global health emergency that articles about the virus started increasing in Canada. The number of articles about COVID-19 showed a sharp increase starting February 2020 for most themes, after the World Health Organization declared it a global health emergency on January 30. Resource shortages and panic buying have been an issue in many countries battling COVID-19 [18]. Our study identified that there were about 300 articles focused on resources. It is postulated that anxiety around sudden lockdowns and uncertainty about the duration of the pandemic might have contributed to the response of preservation of self and family [18]. Retrospectively, it would be beneficial for health care professionals, the government, and the media to work closely together to provide better guidelines and policies for the public, both to reduce anxiety and ensure more equitable distribution of resources.

Additionally, our topic modelling showed that a considerable proportion of news articles in the study focused on the conditions of marginalized populations, such as people experiencing homelessness. Many people experiencing homelessness did not receive timely shelter and space to self-isolate, putting their lives and the lives of others at risk. Thus, our study results highlight the importance of creating an equitable response strategy during future pandemics.

Throughout the course of the pandemic, the most reported information was regarding testing and case reporting. This is consistent with any communicable disease, wherein proper testing, contact tracing, and case reporting are crucial to control the spread of the disease [19]. This information can contribute to increased anxiety, as witnessed by people’s fear of acquiring the disease from health care facilities, and thus being reluctant to access care for other acute illnesses, including heart attacks and strokes [20]. On the other hand, having this information could make people feel more accountable for their actions and encourage them to be more socially responsible. Although the neglect of other conditions was an unintended, unfortunate consequence of pandemic-related public health measures, for future events, more holistic communication from health care professionals (ie, about considering other acute illnesses in times of crisis) and reporting from the media on this topic could aid in better management of people with acute and chronic illnesses.

### Limitations

This study only contains news articles published on CBC's online platform that were tagged with the term “coronavirus.” There are several other sources of media available in Canada and future studies can focus on including multiple different sources of both digital and print media. The pandemic is ongoing and Canada’s response and policies are constantly changing. Thus, doing a long-term study and constantly monitoring multiple media outlets’ efforts will be helpful for future studies. This study nonetheless provides a glimpse of the Canadian media's role in the communication and dissemination of information. The LDA model has certain limitations; for example, the different topics need to be manually interpreted and are open to misinterpretation or overinterpretation. Some of its other limitations include the inability to capture correlations between topics and the use of a fixed number of topics, which must be known ahead of time.

### Comparison With Prior Work

Our study identified several similar and unique themes compared to the themes identified by another similar study on Chinese media reporting [21]. Topics like case reporting, disease spread, medical supplies and resources, and research and development were similarly observed in media in both studies. However, the Chinese study did not identify any themes related to communication from the government or the country's response regarding vulnerable populations. In contrast, although lower in frequency compared to other topics, Canada’s media and response focused on ensuring proper communication from the government and support for vulnerable populations. The government actively communicated with the public, not only through public health officials but also via regular addresses from the country's prime minister during the pandemic. Studies have shown that a leader's address to the public is very effective in reassuring people during times of crisis [22] and the media reports suggest that it played a big part in Canada's response to the pandemic.

Compared to initial communication during the H1N1 pandemic in 2009, which involved the dissemination of misinformation, leading to widespread panic, the slow dissemination of public health information by media outlets initially led to panic early in the COVID-19 pandemic. After the H1N1 pandemic, the Centers for Disease Control and Prevention conducted an audit on public health information dissemination and provided several guidelines for communications in future pandemics [23]. In line with the guidelines, our study topics found that the Canadian response had consistent messaging from federal government and public health officials; however, Canada’s response still fell short with regard to prioritizing marginalized populations and reducing the public’s initial stress stemming from widespread misinformation.

### Conclusions

Our study highlights that, based on the topical analysis of CBC news articles, the Canadian response to the COVID-19 pandemic was a joint effort guided by government policies and communications in conjunction with people’s response and adherence to protocol.

One of the most important factors in preventing the spread of COVID-19 is to empower the public with accurate information [24].

The media plays an important bridging role by relaying information from the government to the public. Thus, by understanding and analyzing the extent to which certain events and policies affect public sentiment and response, policy makers can proactively improve communication for any similar future events, including pandemics, natural disasters, or issues related to national safety.

## Abbreviations

CBC: Canadian Broadcasting Corporation
LDA: Latent Dirichlet Allocation
NLTK: Natural Language Toolkit
